# Supplementary material for: Presence of interictal epileptiform EEG discharges implies increased risk of recurrence after the first unprovoked seizure: Report of the International League Against Epilepsy and International Federation of Clinical Neurophysiology
Source: Clin Neurophysiol Pract. 2025 Aug 28;10:380–91. doi: 10.1016/j.cnp.2025.07.007 (PMC12464601; doi:10.1016/j.cnp.2025.07.007)

**Supplemental Material**

**Supplementary Figure 1:**

**Forest plot of random-effects sensitivity and specificity of interictal epileptiform discharges (IEDs) for seizure recurrence, grouping children and adults separately.**


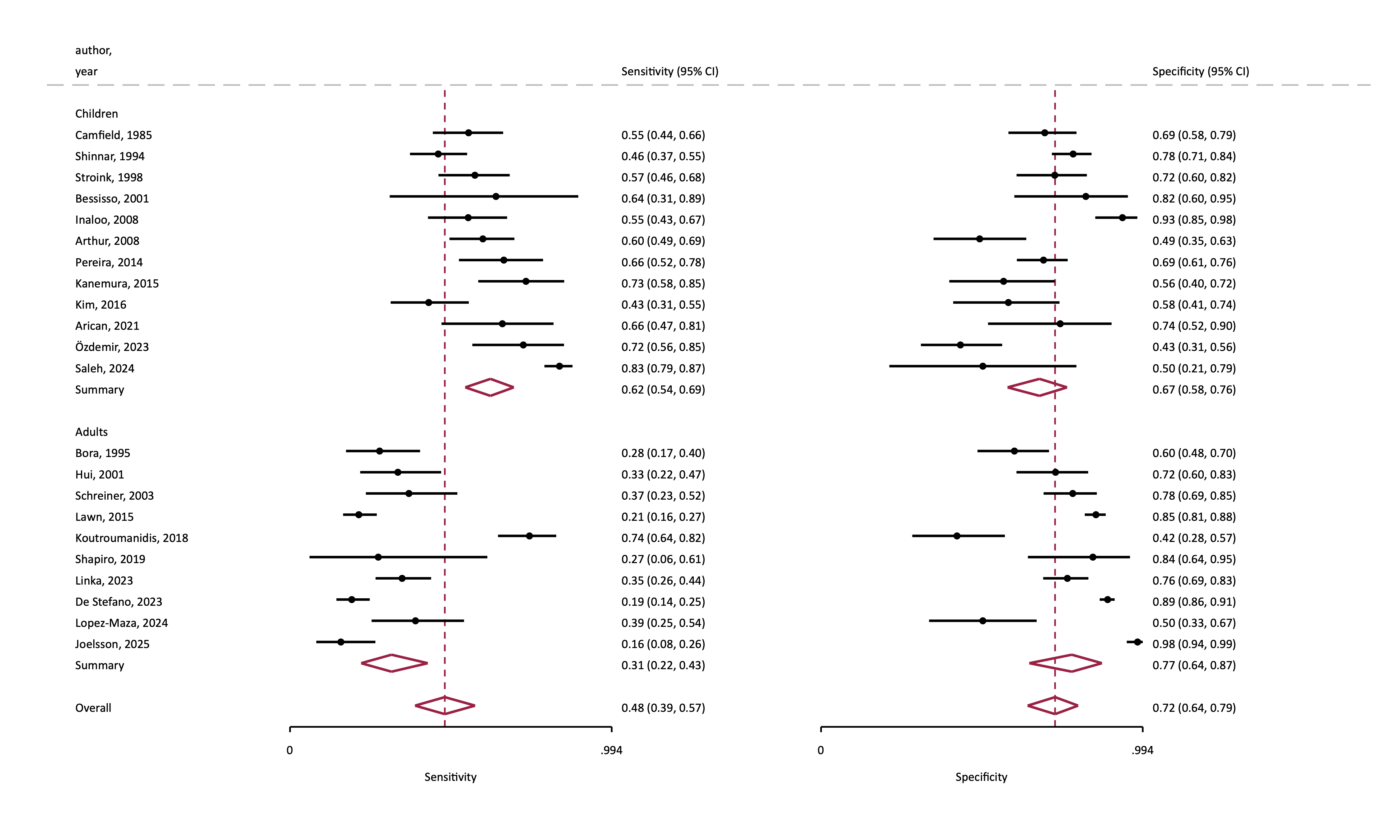


**Supplementary Figure 2:**

**Funnel plot for publication bias and Egger test for effect of small studies**


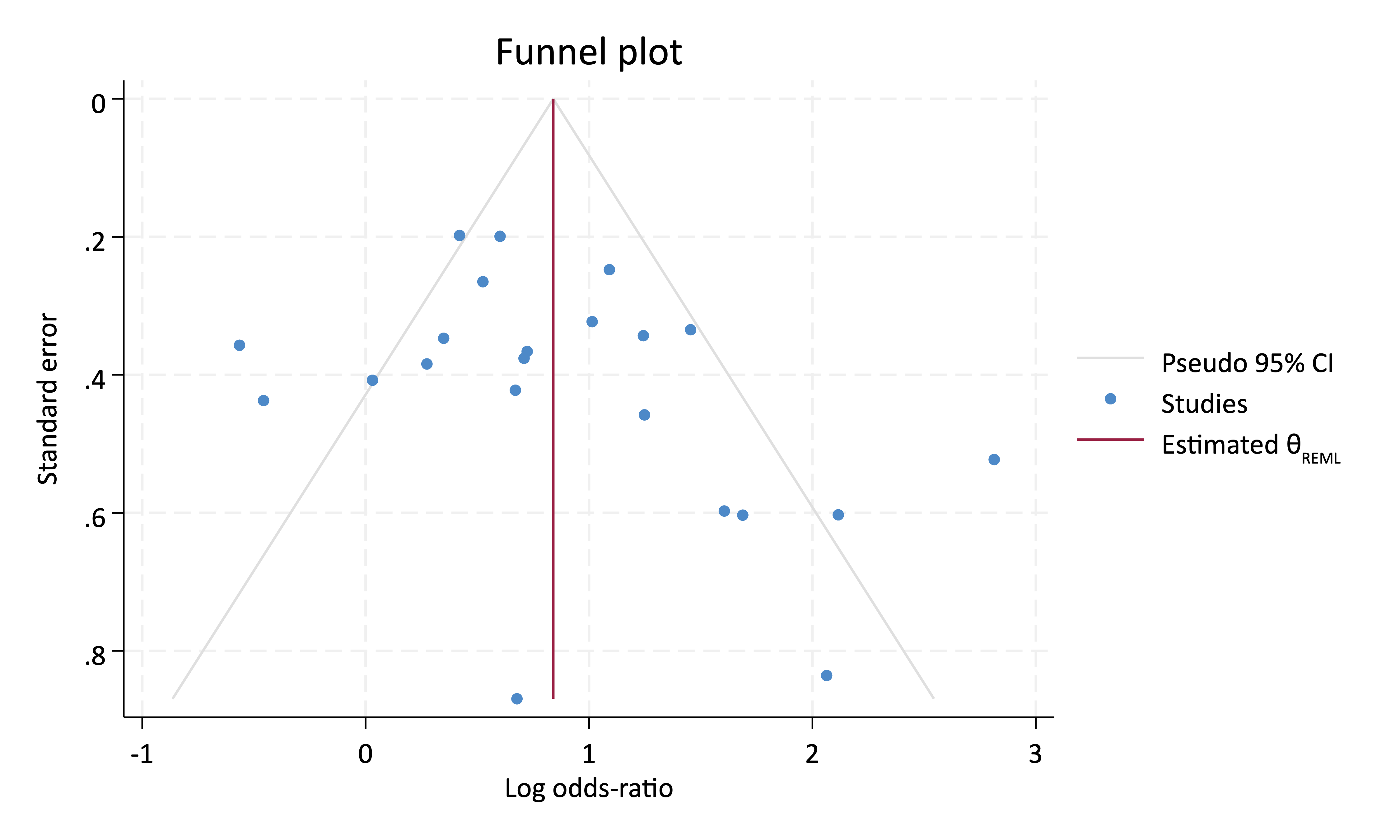


meta bias, egger random(reml)

Effect-size label: Log odds-ratio

Effect size: _meta_es

Std. err.: _meta_se

Regression-based Egger test for small-study effects

Random-effects model

Method: REML

H0: beta1 = 0; no small-study effects

beta1 = **2.11**

SE of beta1 = **1.009**

z = **2.09**

Prob > |z| = **0.0362**

**Supplementary Figure 3:
Meta-regression of age (children vs adult), study design (prospective vs retrospective), year of publication, duration of follow up (years), and influence of some patients receiving ASMs on the predictive ability of EEG for seizure recurrence**

meta regress Pop_child - design – year – followup - ASM_noASM, random(reml)

Effect-size label: Log odds-ratio

Effect size: **_meta_es**

Std. err.: **_meta_se**

Random-effects meta-regression Number of obs = **22**

Method: REML Residual heterogeneity:

tau2 = .**3385**

I2 (%) = **70.25**

H2 = **3.36**

R-squared (%) = **12.45**

Wald chi2(5) = **7.66**

Prob > chi2 = **0.1758**

------------------------------------------------------------------------------

| _meta_es | Coefficient | Standard error | z | P>\|z\| | 95% CI:  lower | 95% CI:  upper |
| --- | --- | --- | --- | --- | --- | --- |
| Pop_child | .6762994 | .3599256 | 1.88 | 0.060 | -.0291418 | 1.381741 |
| design | -.3271406 | .3304806 | -0.99 | 0.322 | -.9748707 | .3205894 |
| year | .0160112 | .0145728 | 1.10 | 0.272 | -.012551 | .0445733 |
| followup | -.0853321 | .1164409 | -0.73 | 0.464 | -.3135521 | .1428878 |
| ASM_noASM | -.0830287 | .3460001 | -0.24 | 0.810 | -.7611764 | .5951191 |
| _cons | -31.55413 | 29.10033 | -1.08 | 0.278 | -88.58973 | 25.48147 |

Test of residual homogeneity: Q_res = chi2(**16**) = 46.11 Prob > Q_res = **0.0001**

**Supplementary Figure 4:**

**Forest plot of the association between interictal epileptiform discharges (IEDs) reported on the initial EEG after first seizure and seizure recurrence, grouping studies with (ASM) and without (no ASM) a proportion of patients being treated with ASM after the first seizure.**


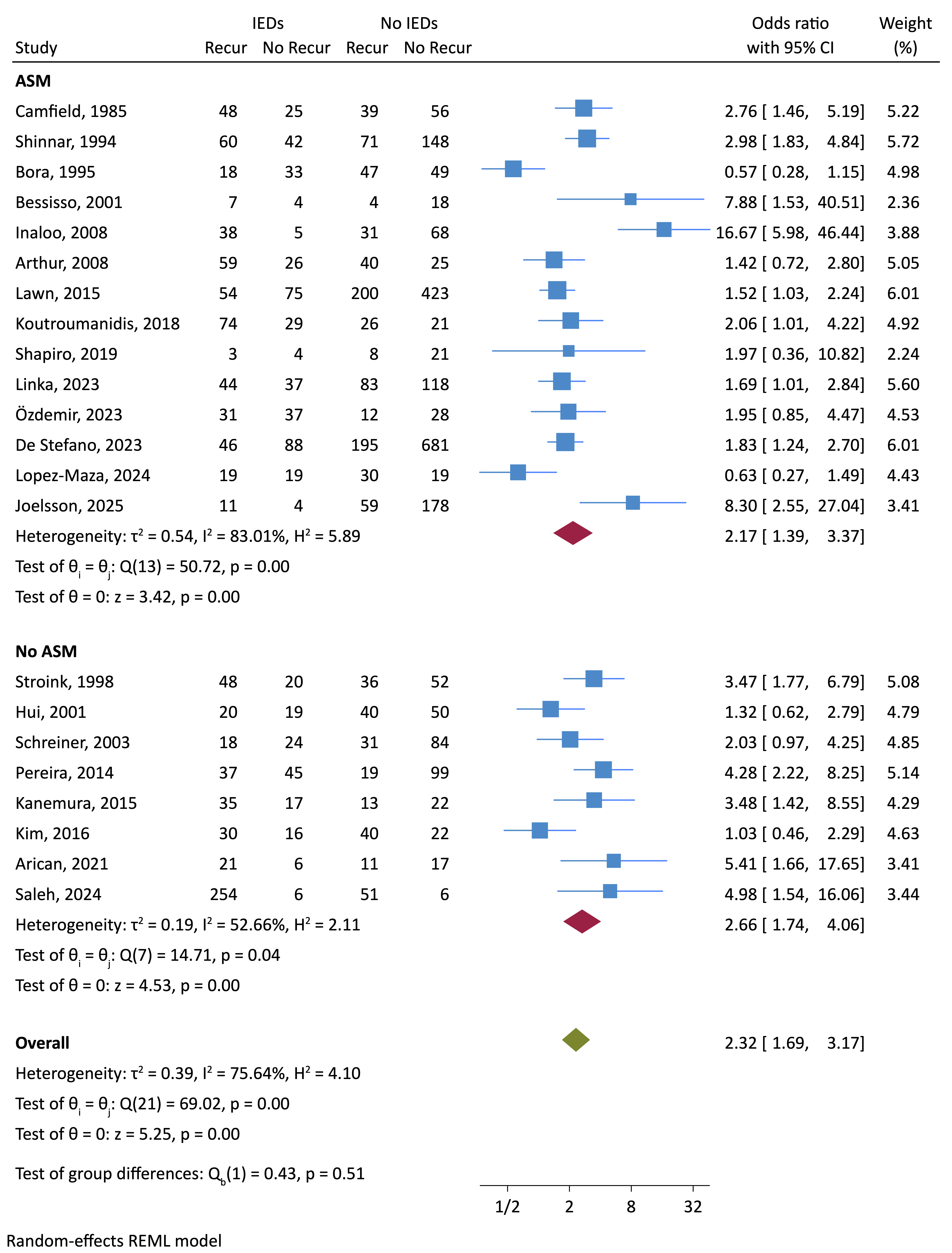

Supplement: Supplementary Data 1 [file mmc1.docx]
